# Preparing research samples for safe arrival at centers and facilities: recipes for successful experiments

**DOI:** 10.1107/S2053230X24006174

**Published:** 2024-07-11

**Authors:** Sarah E. J. Bowman, James Byrnes, Silvia Russi, Christina M. Zimanyi

**Affiliations:** a Hauptman–Woodward Medical Research Institute Buffalo NY14203 USA; b Department of Biochemistry and Department of Structural Biology, Jacobs School of Medicine and Biomedical Science University at Buffalo, State University of New York Buffalo NY14203 USA; c National Synchrotron Light Source II Brookhaven National Laboratory Upton NY11973 USA; d Stanford Synchrotron Radiation Lightsource SLAC National Accelerator Laboratory Menlo Park CA94025 USA; e National Center for CryoEM Access and Training, Simons Electron Microscopy Center New York Structural Biology Center New York NY10027 USA; University of Pennsylvania, USA

**Keywords:** sample preparation, shipping biomacromolecules, structural biology, cryoEM, SAXS/WAXS, X-ray crystallography

## Abstract

Preparation of biomacromolecules for structural biology studies is a complex and time-consuming process. The goal is to produce a highly concentrated, highly pure product that is often shipped to large facilities with tools to prepare the samples for crystallization trials or for measurements at synchrotrons and cryoEM centers. The aim of this article is to provide guidance and to discuss general considerations for shipping biomacromolecular samples. Details are also provided about shipping samples for specific experiment types, including solution- and cryogenic-based techniques. These guidelines are provided with the hope that the time and energy invested in sample preparation is not lost due to shipping logistics.

## Abstract 2

Shipping of biomacromolecules to synchrotrons, cryoEM and sample-preparation facilities is often overlooked and has the potential to erase months and years of hard work. Here, guidelines and procedures are provided to ensure that samples arrive intact and safely at their destination.

## 1. Introduction

Large amounts of time, energy and money are spent on the design, purification and analysis of biomacromolecules. Choosing large user facilities such as a synchrotron or cryoEM center to collect data from your samples is a wise choice as they contain the best tools, equipment and, importantly, the expertise to advance your research. The use of such facilities can be complex at first and requires effort and some patience. Facilities must measure large numbers of samples, maintain throughput, and therefore balance flexibility in operations whilst standardizing operations. Because of this, you, the researcher, will be exposed to many procedures that may seem tedious, but serve a purpose to support your research. Failure to follow certain procedures could delay your measurements, or worse prevent your samples from being measured at all. When you obtain time at a facility, you have an expert (known as your local contact) who will help you through many of the logistical procedures, data collection and analysis. These individuals are excellent scientists and will help you obtain the best-quality data from your samples.

Most facilities provide a ‘mail-in’ (or similar) service that does not require the sample owner to be onsite for measurements, or an ‘in-person’ option, where your samples are either shipped or brought to the facility and measured by the sample owner. Whichever method is employed, the common thread involves shipping or transporting your samples to the facility in a safe, reliable fashion. Given the large amounts of time and energy involved in the preparation of samples, it is important to understand and follow the advice of your local contact when shipping or transporting your samples. Remember, progress towards the goals of your research project cannot happen if the sample is never measured! Here, we discuss shipping/transporting biomacromolecular samples for solution-based and cryogenic techniques, specifically small-angle X-rayscattering (SAXS), crystallization trials and cryogenic samples, to cryoEM or X-ray crystallography facilities.

## 2. General considerations for shipping samples of purified biomolecules

Before data can be collected from a sample, the sample must arrive safely at the facility. Our focus here is to provide general advice for the transport of samples between your home laboratory and a remote data-collection resource. Some principles, such as making use of secondary containment and maintaining communication with local contacts, are universally applicable. Other details will be specific to the facility that you are making use of, for example precise labeling instructions and biosafety rules. Each facility should be consulted for specific standard operating procedures (SOPs) and requirements. Biological laboratories must comply with local, state and national regulations that may vary in different locations, and the transport between the locations may be regulated. For example, in the United States, the transport of biological samples across state lines is regulated by the US Department of Transportation (DOT) and International Air Transport Association (IATA). Even if your sample is not a hazardous or regulated material, including a manifest listing all contents of the shipment in plain language will prevent any confusion and help to avoid delays or potentially large fines. Many university departments offer training and assistance in shipping biological and other hazardous materials (including dry ice) through environmental health and safety (EH&S) offices.

For both successful commercial shipping and productive experiments, general principles on packaging and labeling should be followed. Small tubes should always be placed in a larger secondary container; for example, tubes holding 2 ml in volume or less should be placed into larger conical tubes, cardboard or plastic boxes, or sealable plastic bags. If the tubes are too small to label clearly, the secondary container should be labeled, and non-identical samples should be in separate containers. Adhesive labels often fall off at low temperatures and should be avoided. Permanent pens should be used to label tubes to ensure labels do not wipe off. Any paper labels or forms included in shipments should be protected in plastic bags. Each facility will have their own requirements for sample labeling, and you should be aware of these and ask for help from your local contact if they are not clearly provided to you.

A key logistical consideration is the correct box and shipping speed. Overnight shipping should be used whenever possible. Shipping and receiving offices are generally closed at weekends at many facilities. Therefore, plan to have samples arrive before the weekend. It is best practice to anticipate shipping delays. It is the experience of the authors that shipping delays do happen, especially amongst poorly packed boxes, packages going through customs, weather-related events *etc.* Thus, packing your samples in the right box is critical. Use insulated boxes, and to avoid delays when shipping on dry ice follow all instructions of the shipping carrier as well as your institution’s safety office (*i.e.* the use of cardboard overpack around Styrofoam and proper hazardous materials labeling).

To make sure that your samples are well taken care of upon arrival, you should provide shipping information such as tracking numbers to your local contact. Along with the inventory that you provide, storage requirements (whether 4°C, −20°C or −80°C) should be clearly indicated. Both a printed and a digital copy of the package inventory should be provided.

Once your samples safely arrive at an instrumentation facility, it is important that biomolecules in solution are still in an appropriate state for data collection. The stability of your samples under shipping conditions should be tested before assuming that they are ideal for your sample of interest. Several key tests involve the following.

(i) Leaving the sample at room temperature and 4°C for several hours to determine whether precipitates form.

(ii) Perform a freeze–thaw cycle.

(iii) Running samples from steps (ii) and (iii) through a size-exclusion chromatography (SEC) column (or equivalent analytical method) to determine the quantity of oligomers.

(iv) Attempt lyophilization.

The results of these tests will help you decide how to ship your samples (at either 4°C or frozen). Shipping on dry ice ensures that samples will remain frozen for the duration of transport, but buffers may experience substantial pH changes due to CO_2_ absorption, so it is important to let samples and buffers remain at −80°C for some amount of time off of dry ice before thawing (Murphy *et al.*, 2013 ▸). If the sample partially aggregates after a freeze–thaw, you should inquire about the availability of an SEC column for a final polishing step at the final facility. This and similar scenarios should be discussed with your local contact well in advance to make the right plans.

## 3. General consideration of samples for solution-based measurements

**Figure 1:**
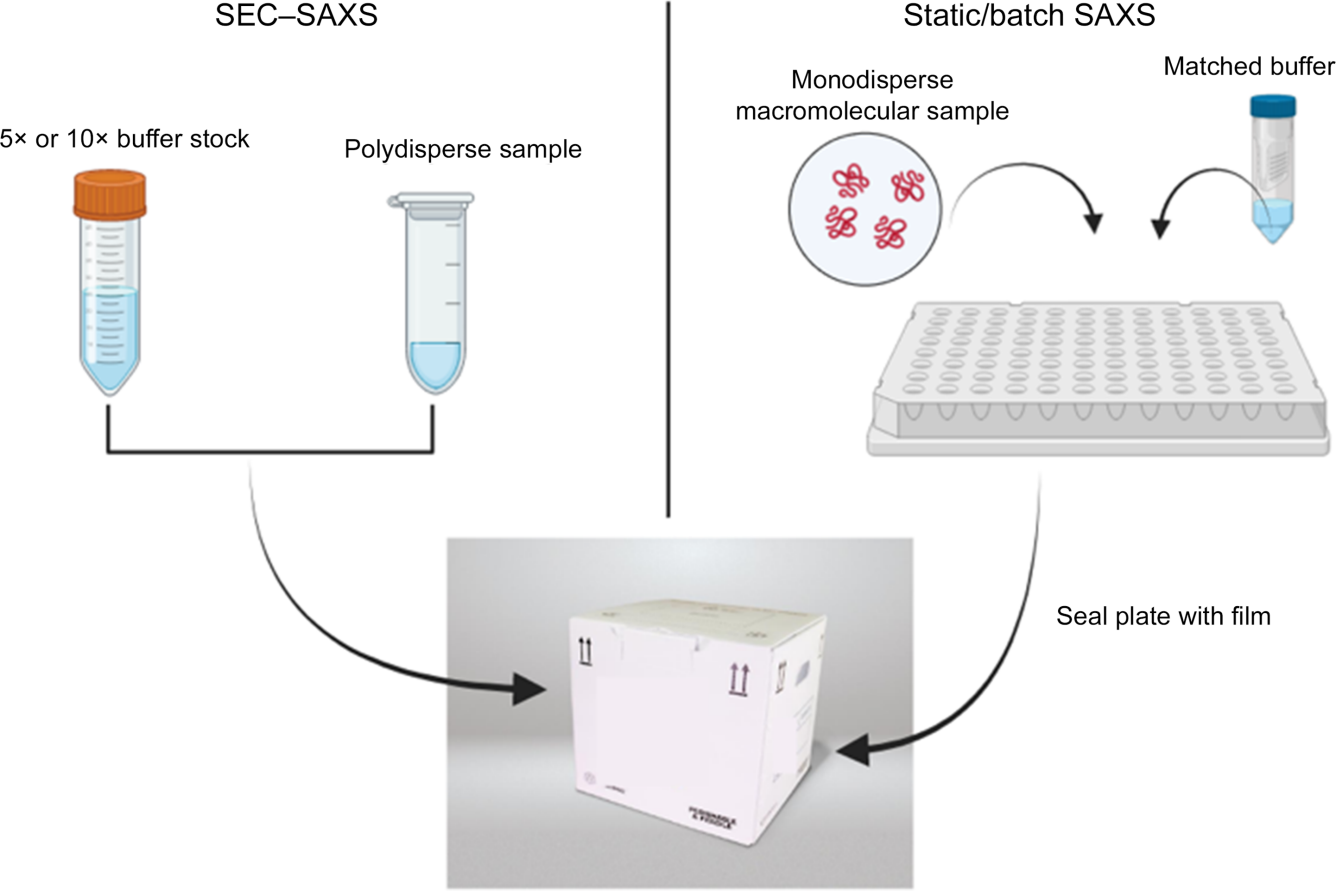
Typical format for solution-based X-ray scattering experiments of biological macromolecules. For samples that are pure and monodisperse, samples are generally loaded into a plate (right panel; check with your facility for exact formats). Matched buffer from either the concentrator eluate, purification process or dialysate must be shipped with the sample. Plates should be sealed with film and a lid. If samples are polydisperse, SEC–SAXS can be employed (left panel) and requires running buffer to be shipped along with samples. Include a 5× or 10× stock of buffer to be diluted at the facility. Solution samples should be packed carefully and in a reusable thermal shipper (bottom) that can hold temperature for several days.

There are many excellent reviews that discuss the sample preparation and optimization of solution-based measurements (Jeffries *et al.*, 2016 ▸; Graewert & Jeffries, 2017 ▸). However, based on the authors’ knowledge, the shipment of samples is not discussed in detail in the literature and will be addressed here. When shipping solution-based samples for SAXS be sure to follow the stability characterization steps described above. These results will determine whether you have a monodisperse sample suited for batch SAXS measurements, or whether a size-exclusion chromatography coupled with small-angle X-ray scattering (SEC–SAXS) experiment will be needed to isolate aggregates and/or oligomers. Clarify the necessary format with your local contact, as the reagents and shipping formats are different (Fig. 1 ▸). Ideally, samples for SAXS should be shipped at 4°C and measured fresh, but this is not always possible. Often, samples are shipped in 96-well plates and stored at the facility for measurement later (Yang *et al.*, 2021 ▸; Classen *et al.*, 2013 ▸). For SEC–SAXS, you will need to ship plenty of running buffer, necessary to equilibrate the column, run the samples and account for any issues that may arise. For example, a beam dump might occur and require a wait before measurements can take place again. It is not optimal to stop the flow on a sizing column and therefore buffer will continue to flow during the beam-recovery process. Thus, sending excess running buffer is an inexpensive step and prevents further interruptions to the workflow. When shipping, a 5× or 10× stock to be diluted at the facility is ideal and reduces the volume/weight of the shipping box. The container should be a conical tube and if shipping frozen, account for headroom so that the tube does not crack and leak into the shipping container. Avoid glass, and if 1× stocks of buffer are shipped in large volumes, plastic bottles are ideal. It is important to ship both the sample and buffer in the same state to account for component changes during the freezing process (such as states of reductant). Moreover, the choices for ideal buffer conditions should be made during the sample-preparation process, which is not discussed in this article.

Purchasing a reusable thermal shipper can insulate you from lost samples because of delays. Several types exist on the market, so be sure to chose one that is reusable, contains an inner insulating box and a multi-use external shipping box that can be replaced if needed (Fig. 1 ▸). Many come with inner boxes that are actually thermal packs and are placed at the appropriate temperature before shipping. These are rated to keep samples at temperature for a length of time (usually days) and are worth the money, given the time and energy spent on preparing the sample.

When measuring samples onsite or mailing in, remember to send excess buffer and additional sample volume if possible. After the results are generated, it can be useful to design new experiments (such as varying concentration) within the same allocated beamtime. Thus, providing extra can maximize the beamtime and improve the results.

## 4. General considerations for shipping samples for crystallization

There are numerous resources that address methods for making biomolecular crystals (Chayen & Saridakis, 2008 ▸; McPherson & Gavira, 2014 ▸; Budziszewski *et al.*, 2023 ▸), as well as describing techniques to successfully harvest crystals for data collection (Senda *et al.*, 2016 ▸; Wright *et al.*, 2021 ▸; Fischer, 2021 ▸). There is a limitation in the literature, however, regarding shipping solution samples to centralized crystallization facilities for assistance with the crystallization process; this gap is addressed here. The goal in a crystallization experiment at a facility is often twofold: (i) to find initial crystallization conditions for your biomolecular sample and (ii) to optimize from the conditions found in step (i) or from other known conditions. Crystallization requires your biomolecular sample to form an ordered lattice, but to not precipitate and denature; therefore, the sample needs to be stable and soluble until crystals nucleate. Specific considerations to increase the chances of crystallization include buffer components and sample concentration. Generally, all components that keep the sample stable should be included, including buffers, salts, glycerol and detergents for membrane-protein samples. However, if it is at all possible, the buffer, salt and glycerol concentrations should be kept as low as possible. Phosphate buffers often form insoluble salts (and crystallize). Glycerol is a solubilizing agent; at concentrations higher than ∼5% in the final crystallization drop it can limit crystallization. The best buffer is the simplest formulation possible that maintains solubility, homogeneity, stability and activity of the sample. For membrane proteins, detergents are typically used at 1.5–2× the critical micelle concentration for crystallization. An appropriate sample concentration can be determined using the Hampton Research Pre-Crystallization Test (PCT). If you would like the facility to perform a PCT for your sample, be sure to be in touch with your local contact prior to shipping and to include extra buffer in case the sample needs to be diluted.

**Figure 2:**
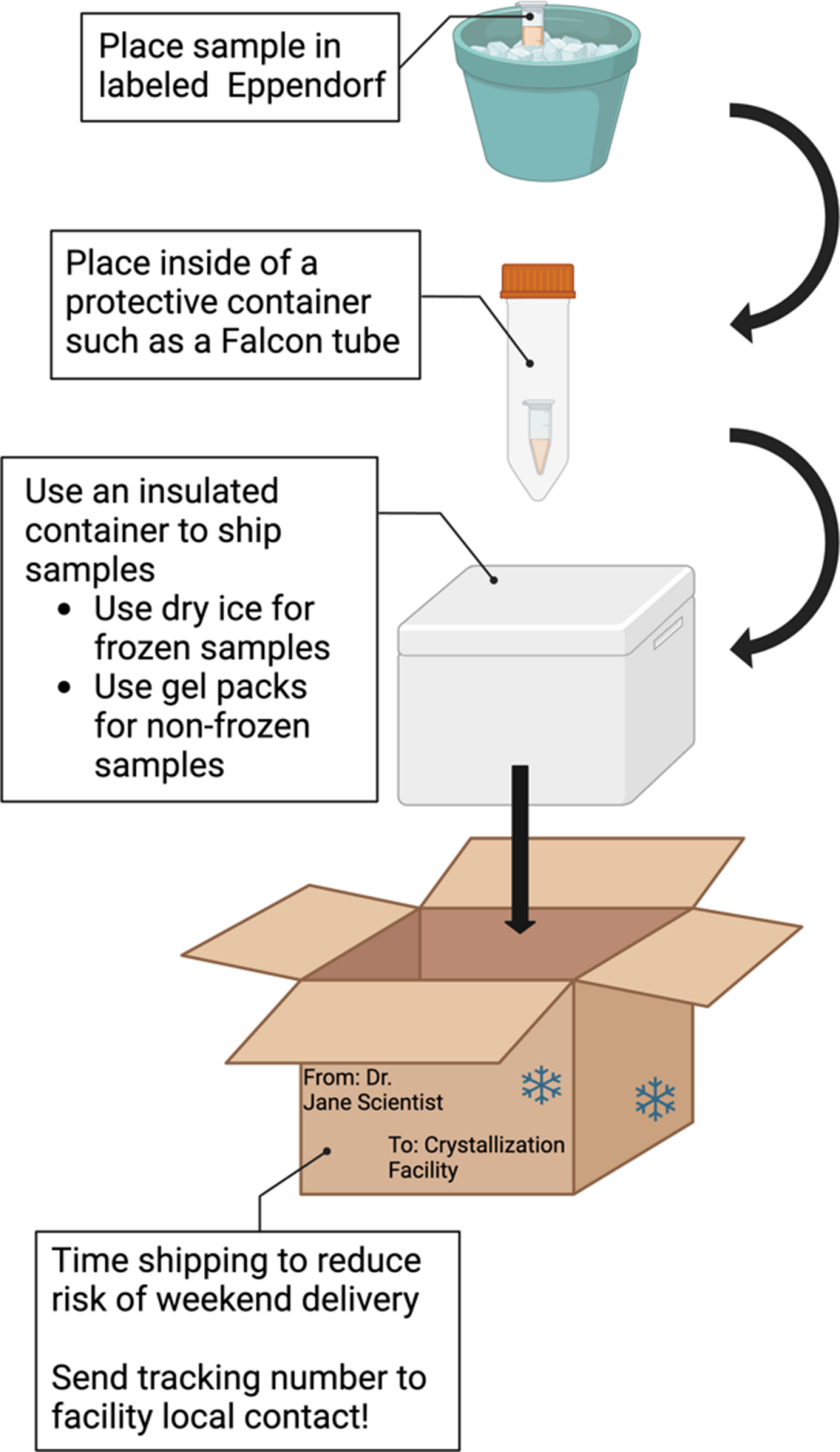
For crystallization experiments, samples should be packed into a clearly labeled 1.5 ml tube placed inside a protective container. Ensure that the tube is sealed and protected against any potential leaks. An insulated Styrofoam shipping container is recommended with dry ice for frozen samples or gel packs to maintain the sample temperature. Alternatively, a Credo Cube can be used to ship solution samples. Shipping overnight Monday to Wednesday helps to reduce the risk of a weekend delivery delay. When the package is shipped, send an email with the tracking number to the facility.

Shipping solution biomolecular samples for crystallization trials requires similar stability investigations as those needed for SAXS. Firstly, determine the best temperature at which your sample remains stable, including testing that the sample remains well folded and stable after a freeze–thaw cycle. An additional question is how many freeze–thaw cycles the sample can handle. If the sample is only stable for one freeze–thaw cycle, make sure to aliquot the sample into small volumes to facilitate multiple crystallization trials for optimization experiments. If the sample is frozen, ship on dry ice or in a charged dry shipper. If the sample cannot be frozen, ship with gel packs or use a thermal shipper. It is safest to place the sample in a well labeled Eppendorf tube or set of tubes, then place the tube(s) into a protective container such as a Falcon tube (Fig. 2 ▸).

## 5. General considerations for shipping cryogenic samples

**Figure 3:**
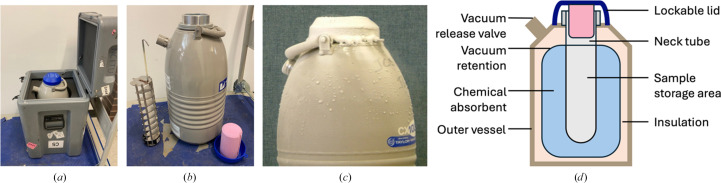
Dry-shipping dewars are used to transport cryogenic samples. (*a*) A hard-shell insulated box holds the shipping dewar. (*b*) The dewar can hold a sample-transport device for high-capacity pucks (left) or 50 ml tubes can be placed directly into the storage area. The dewar also has a lockable lid (right with pink cap). (*c*) When performing a vacuum-failure test, any frosting or condensation observed on the outside of the dewar indicates a vacuum failure. This dewar should not be used for shipping. (*d*) Critical components are indicated in a cartoon schematic of a dewar cross-section.

Cryogenic samples, such as macromolecular crystals or cryoEM grids, are shipped to facilities in liquid-nitrogen dry-shipping dewars. Inside the outer vessel, the dewars have a liquid nitrogen-absorbing material and an insulating layer. A high vacuum is maintained to provide very good thermal insulation between the inside and the outside of the dewar (Fig. 3 ▸).

To avoid sample loss when shipping under cryogenic conditions, it is very important to inspect the dry-shipping dewars at least once every 3–6 months, and ideally before each use. A fully functional dry shipper can keep samples frozen for 14+ days, but over time there is a degradation in performance that can be attributed to vacuum loss, the accumulation of moisture in the absorbent material, or damage or loss of the absorbent material. There are three main tests that must be performed on any dry-shipper dewar: vacuum failure test (Fig. 3 ▸*c*), absorbent material test and insultation test, briefly outlined in Table 1 ▸ (a full description of how to perform these tests can be found at https://smb.slac.stanford.edu/facilities/hardware/cryotools/shipping-dewar-testing.html or https://www.diamond.ac.uk/Instruments/Mx/Common/Common-Manual/Shipping-Samples/Care-of-dry-shippers.html.)

When preparing the dewar for shipping, always charge it as recommended by the manufacturer and ensure that all residual liquid nitrogen is poured out of the dewar before shipment. Minimize the spillage of liquid nitrogen on the vacuum-release valve near the top of the dewar.

Dewars should be fully dried between uses. Recharging the absorbent material before it dries leads to degradation of the material. Drying can be accelerated by purging with air or dry nitrogen. It may take several days to properly dry the shipping dewar. Regular measurement of the dewar weight compared with the initial warm and dry weight is the best method to ensure that it is dry.

## 6. Samples for X-ray crystallography experiments

Harvesting macromolecular crystals is a delicate and time-consuming task, and it is very important to select sample holders (magnetic pin bases) and sample containers (cassettes, Uni-Pucks, ALS Pucks *etc.*) that are compatible with the robotics at the synchrotron site that you are shipping the samples to before proceeding. Your local contact can help you in providing this information, or you can visit https://smb.slac.stanford.edu/facilities/hardware/SAM/robosync/ for a list of worldwide synchrotron beamline robotics and compatibility.

Proper sample-pin preparation is also essential for a successful experiment. Microtubes should be secured in the sample-pin bases with epoxy. Any epoxy with a cure time of between 5 min and 24 h should work well. Superglue and superglue gel are less reliable than epoxy for affixing microtubes. The use of other types of adhesives such as wax, nail polish and Duco cement should be avoided. Be careful not to apply excess epoxy to the pin base or post pin, and do not apply grease or excess cryoprotectant to the pin body.

If you wish to mark your sample pins with different colors, use only permanent marker. Paint or nail polish should not be used as they may change the shape of the pin or be sticky. Cryogenic color dots for 1.5 ml microtube tips should also be avoided, as they may become partially or completely detached from the pin base and interfere with the sample mounting on the goniometer head. If you reuse your pins, inspect them for corrosion and loose microtubes, as these can interfere with proper sample mounting on the goniometer.

Finally, special care must be taken when loading the sample containers into a shipping dewar. Be careful not to drop them, as this could damage the neck of the dewar. Never pack items that are too large, or incorrectly place the lid to make the items fit. Forcing the lid down may damage the neck. The shipping canister used for shipping pucks and Uni-Pucks has a locking bar to hold the pucks in place during shipping; without this bar, the pucks may become stuck in the shipping dewar and be impossible to recover under cryogenic conditions, leading to the loss of all samples in the dewar.

## 7. Samples for cryoEM experiments

CryoEM grids must remain below −160°C (the temperature of vitrified ice) after freezing or they will become unsuitable for data collection. A dry-shipping dewar, as described above, is sufficient to transport cryoEM grids and is our recommended method. All of the general shipping dewar precautions and considerations detailed above should be followed.

CryoEM is often used to study infectious agents such as viruses and pathogenic bacteria. Although the samples are frozen, they could thaw and thus all biosafety considerations still apply to biological specimens frozen on cryoEM grids. Not all facilities accept samples designated BSL-2 or above due to compliance at the specific location. Significant effort can be required to allow such samples (Sherman *et al.*, 2013 ▸). If working with samples designated BSL-2 or higher, it is essential to follow the SOPs and guidelines provided by the center.

**Figure 4:**
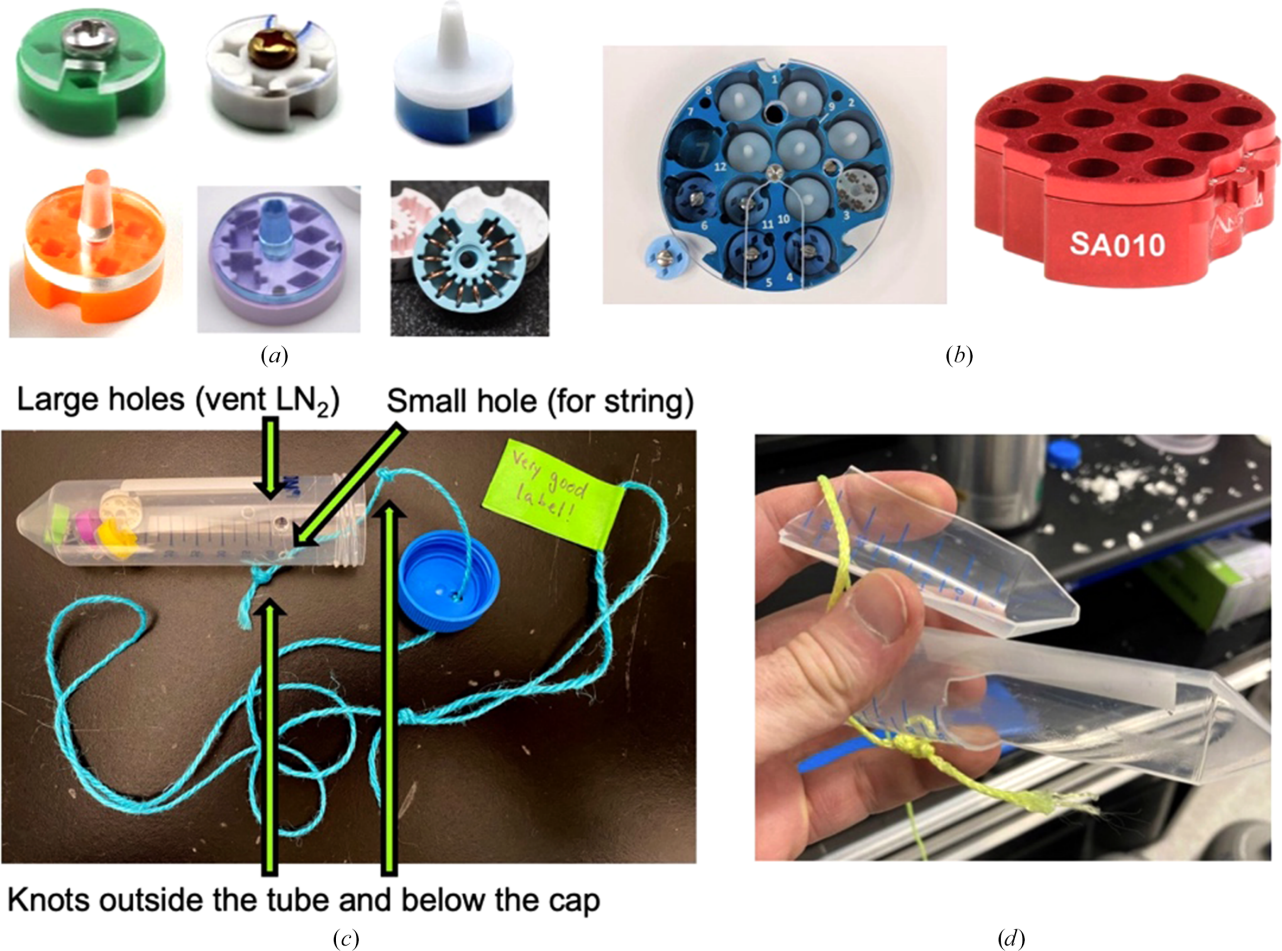
Standard cryoEM sample-storage and shipping solutions. (*a*) Grid boxes hold four or more grids per box with lids that can be tightly secured to ensure that grids do not fall out. (*b*) High-capacity ‘pucks’ can be stored in liquid nitrogen and are useful for sample organization. (*c*) Grid boxes can be put into 50 ml conical tubes stored in liquid nitrogen. Vent holes and a hole for a string to retrieve the tube from a dewar should be drilled into the sides of the tube. At least two knots, one outside the tube and one below the cap, should be tied so that the tube can be safely retrieved. (*d*) If vent holes are not drilled into the tube it can explode, causing safety issues and lost samples.

Frozen grids are small and fragile and thus are stored in a grid box (Fig. 4 ▸*a*). Most vendors sell grid boxes that hold up to four grids, although expanding product offerings and custom 3D printing solutions have allowed a multitude of possibilities (Hamaguchi & Yonekura, 2020 ▸). Multiple grid boxes can be combined into a secondary container (either a high-capacity ‘puck’ or a 50 ml conical tube; Figs. 4 ▸*b* and 4 ▸*c*; Scapin *et al.*, 2017 ▸). For shipping, this secondary container then goes into a charged dry shipper.

Electron microscopes at large cryoEM centers use robotic grid loaders that may require the grids to be secured into a cassette for loading. You should verify with each center what their grid-submission requirements are, because they are not the same at all centers. Universally, however, all cryo-grids are stored in grid boxes that are secured safely with lids. Grid-box lids should be tightly secured so that the grids do not fall out of the box during transit. Grids that have fallen out of boxes are often not recoverable and if multiple grids have fallen out the identity of the sample may be unknown. Grid boxes should also be clearly labeled with useful labels. You should verify with the center that you are shipping to whether there are specific labeling requirements. If you are shipping more than one box, consider using boxes of different colors to make them easily distinguishable.

Once your grids are safely secured in well labeled boxes with tight lids, you should make sure that they are in a good secondary container. The two most common secondary containers are 50 ml conical tubes with strings attached or ‘pucks’ (Fig. 4 ▸*b*). While conical tubes are significantly cheaper, the puck system is more organized and makes a mix-up of boxes less likely. Some centers prefer to receive pucks, while others prefer conical tubes, so if you have both options find out which is preferred at the center that you are using.

There are safety considerations for storing grids in 50 ml conical tubes. Before being placed into a dry shipper, they are usually stored in a liquid-nitrogen storage dewar. Liquid nitrogen expands roughly 700 times in volume when it boils into gas. To avoid exploding conical tubes (Fig. 4 ▸*d*), punch holes in the lid and sides of your tubes. You will also need holes in the tube to attach a string for retrieval of the tube from the storage or shipping dewar (Fig. 4 ▸*c*). When shipping, you will also want to prevent jostling of the grid boxes in the tube. A few tips to do this include stuffing the tubes with tissues (for example Kimwipes). You can also add empty conical tube bundles to the dry shipper to keep a single tube from moving too much.

Finally, it is important to consider how many grids you are sending and, if you intend to collect data for high-resolution data collection, whether they need to be screened. Different centers will have different policies for how many grids can be submitted per session and what the pre-screening requirements are. Also, ‘screening’ in cryo-EM does not has a single definition, so you should be sure to ask what the center expects and means by ‘pre-screened’ grids.

## 8. Conclusions

When preparing a shipment, consider that someone who has never seen your samples will unpack it. Therefore, make sure that all labels are clear and communicate the expected item inventory to your local contact. Ensure that any SDS, DOT and facility documentation necessary arrive with your samples to prevent safety violations that could result in sample damage. Plan well in advance for international shipments and discuss with your local contact any import/export controls that need attention before measurement. Spending time addressing the logistics of sample shipping and transport mentioned in this article will help to ensure better experimental results and a positive experience. The staff scientists helping to collect your data hope that your experiment will be successful and are happy to answer your shipping questions.

## Figures and Tables

**Table 1:** Dry-shipping dewar maintenance testing protocols

Type of test	Steps
Quick verification of dewar function	(i) Charge the dewar exactly as intended for shipping samples.
	(ii) Leave for the duration of shipping (*i.e.* 2–3 days). Check after the last day and verify that the shipper is still at cryogenic temperature.
	
Vacuum failure test	(i) Charge the dewar exactly as intended for shipping samples.
	(ii) Wipe the outside of the dewar dry with a cloth.
	(iii) Wait 2–3 h.
	(iv) Inspect the outside of the dewar for condensation. If condensation is apparent, the vacuum has failed. Do not use this dewar.
	
Absorbent material test	(i) Measure the weight of the dry and empty dewar at room temperature without the lid (‘dry weight’).
	(ii) Saturate absorbent material with liquid nitrogen.
	(iii) Pour out excess nitrogen.
	(iv) Reweigh the charged dewar without the lid. The weight difference is the mass of stored nitrogen and should be ∼3 kg.
	(v) Perform this test periodically and compare the values over time.
	
Insulation test	(i) Charge the dewar and remove excess liquid nitrogen. Leave the dewar capped.
	(ii) Weigh the dewar after ∼1 h.
	(iii) Repeat the measurement after 24 h.
	(iv) Calculate the mass loss between measurements (the mass loss for a new dry shipper should be around 150–200 g per day depending on model).
